# Supplementary material for: Cytochrome c lysine acetylation regulates cellular respiration and cell death in ischemic skeletal muscle
Source: Nat Commun. 2023 Jul 13;14:4166. doi: 10.1038/s41467-023-39820-8 (PMC10345088; doi:10.1038/s41467-023-39820-8)
Supplement: Supplementary file 1 — Supplementary Information [file 41467_2023_39820_MOESM1_ESM.pdf]

Supplementary Information for

Cytochrome *c* Lysine Acetylation Regulates Cellular Respiration and Cell Death in Ischemic Skeletal Muscle

Paul T. Morse[1,†], Gonzalo Pérez-Mejías[2,†], Junmei Wan[1], Alice A. Turner[1,3], Inmaculada Márquez[2], Hasini A. Kalpage[1], Asmita Vaishnav[3], Matthew P. Zurek[1,3], Philipp P. Huettemann[1], Katherine Kim[1], Tasnim Arroum[1], Miguel A. De la Rosa[2], Dipanwita Dutta Chowdhury[3], Icksoo Lee[4], Joseph S. Brunzelle[5], Thomas H. Sanderson[6], Moh H. Malek[7], David Meierhofer[8], Brian F.P. Edwards[3], Irene Díaz-Moreno[2,*], and Maik Hüttemann[1,3,*]

[1] Center for Molecular Medicine and Genetics, Wayne State University, Detroit, MI 48201, USA;
[2] Instituto de Investigaciones Químicas, Universidad de Sevilla - CSIC, 41092 Sevilla, Spain;
[3] Department of Biochemistry, Microbiology, and Immunology, Wayne State University, Detroit, MI 48201, USA;
[4] College of Medicine, Dankook University, Cheonan-si, Chungcheongnam-do 31116, Republic of Korea;
[5] Life Sciences Collaborative Access Team, Northwestern University, Center for Synchrotron Research, Argonne, IL 60439, USA;
[6] Department of Emergency Medicine, University of Michigan Medical School, Ann Arbor, MI 48109, USA;
[7] Department of Health Care Sciences, Eugene Applebaum College of Pharmacy & Health Sciences, Wayne State University, Detroit, MI 48201, USA;
[8] Max Planck Institute for Molecular Genetics, 14195 Berlin, Germany;
† These authors contributed equally to this paper.
* Correspondence: mhuttema@med.wayne.edu; Tel.: +1-313-577-9150; Fax: +1-313-577-5218 (Maik Hüttemann) and idiazmoreno@us.es; Tel.: +34-954489513; Fax: +34-954460065 (Irene Díaz Moreno)

Contents

Supplementary Figures 1 to 13

Supplementary Tables 1 and 2

a

```
refinement {
 refine {
  occupancies {
   constrained_group {
    selection = chain A and resseq 39 and (name OH or name CH or name CH3)
   }
   constrained_group {
    selection = chain B and resseq 39 and (name OH or name CH or name CH3)
   }
   constrained_group {
    selection = chain C and resseq 39 and (name OH or name CH or name CH3)
   }
   constrained_group {
    selection = chain D and resseq 39 and (name OH or name CH or name CH3)
   }
  }
 }
}
```

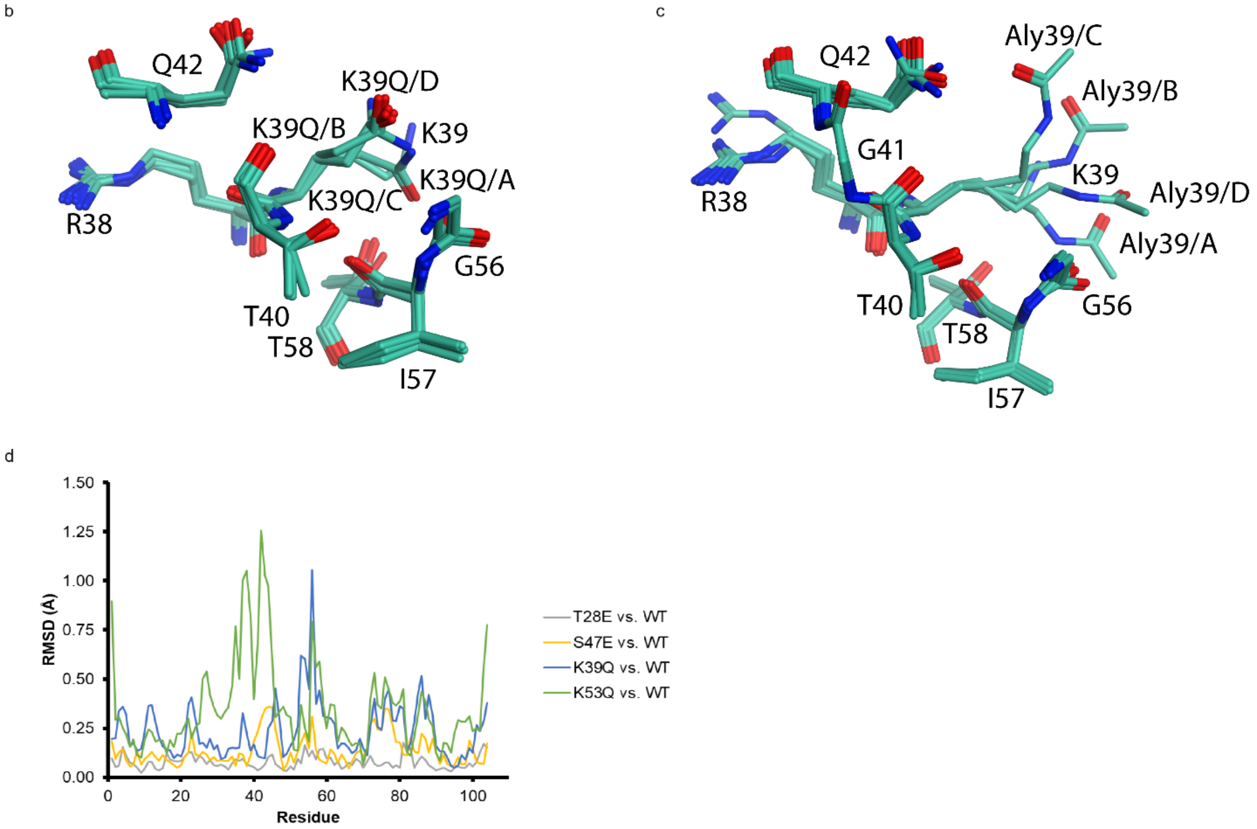

Supplementary Figure 1. Crystal structure differences from WT. (A) Phenix.Refine parameter code, kindly provided by Dr. Pavel Afonine, used to individually refine occupancies for each acetyl group of Aly39 crystal structure (8DVX). **(B)** The four molecules of the K39Q crystal structure (8DZL) overlayed with molecule A in the rat WT structure (5C0Za) with all nearby residues within 3.5 Å. **(C)** The four molecules of the Aly39 crystal structure (8DVX) overlayed with molecule A in the rat WT structure with all nearby residues within 3.5 Å. **(D)** The respective backbone RMSD values for molecule A of T28E (5DF5a), S47E (6N1Oa), K39Q (8DZLa), and K53Q (7LJXa) compared to molecule A in the rat WT structure (5C0Za).

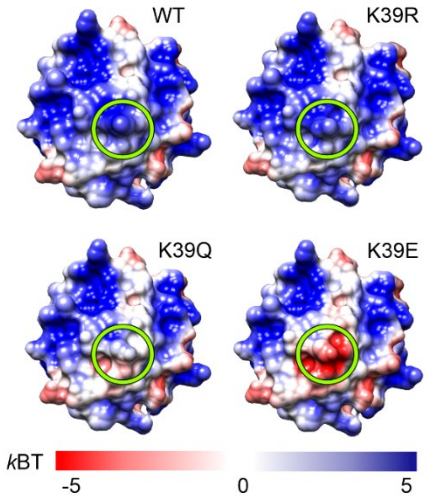

Supplementary Figure 2. Electrostatic surface potentials of WT and mutant Cyt*c* species. Electrostatic potentials were calculated at 150 mM ionic strength. Green circles denote mutated residues at residue 39.

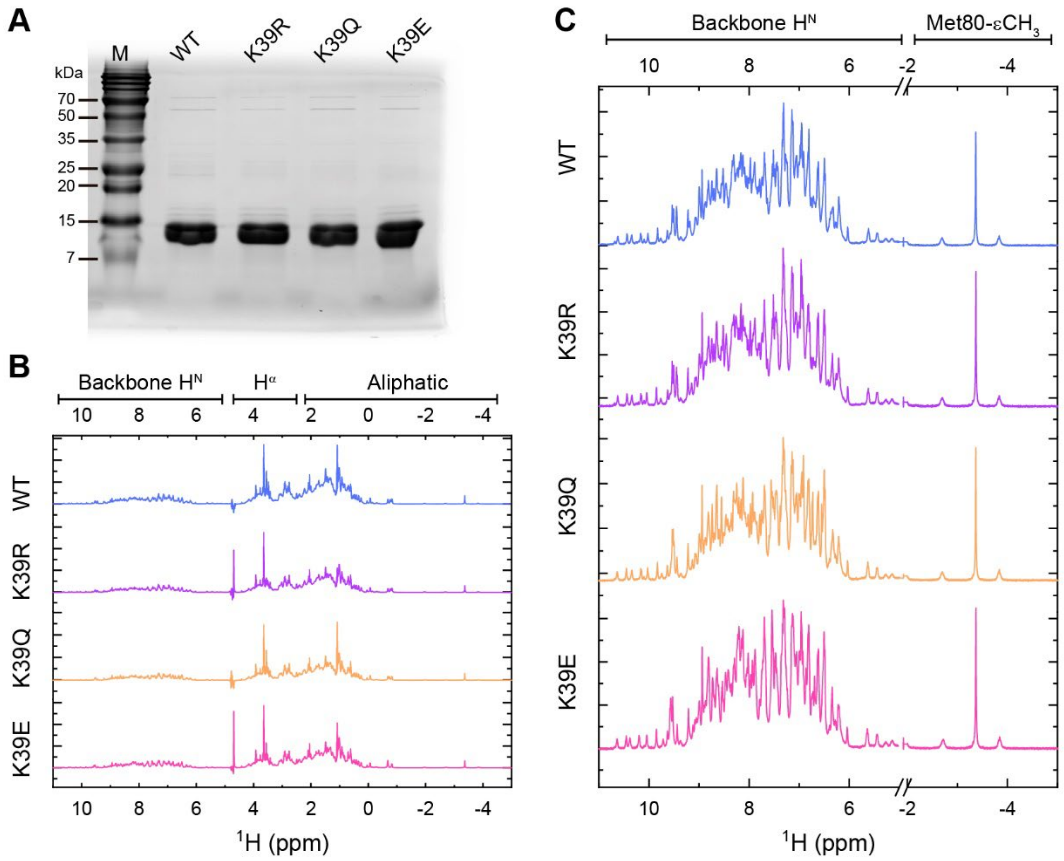

Supplementary Figure 3. Quality control of recombinant Cyt*c* samples. (A) 15% SDS-PAGE gel of ^{15}N-labelled mouse recombinant Cyt*c* proteins. A total of 5 µg of protein for each sample was loaded onto the gel, and it was stained for protein detection with 0.25% Coomassie Brilliant Blue R-250 in 45% methanol and 10% acetic acid. M: molecular weight marker (n = 2). **(B)** Complete 1D ^{1}H NMR spectra of reduced ^{15}N-labelled Cyt*c* (WT in blue, K39R in purple, K39Q in yellow and K39E in pink). **(C)** Detailed view of the backbone amide and heme axial ligand M80-εCH_3 signals of the 1D ^{1}H NMR spectra. Source data are provided as a Source Data file.

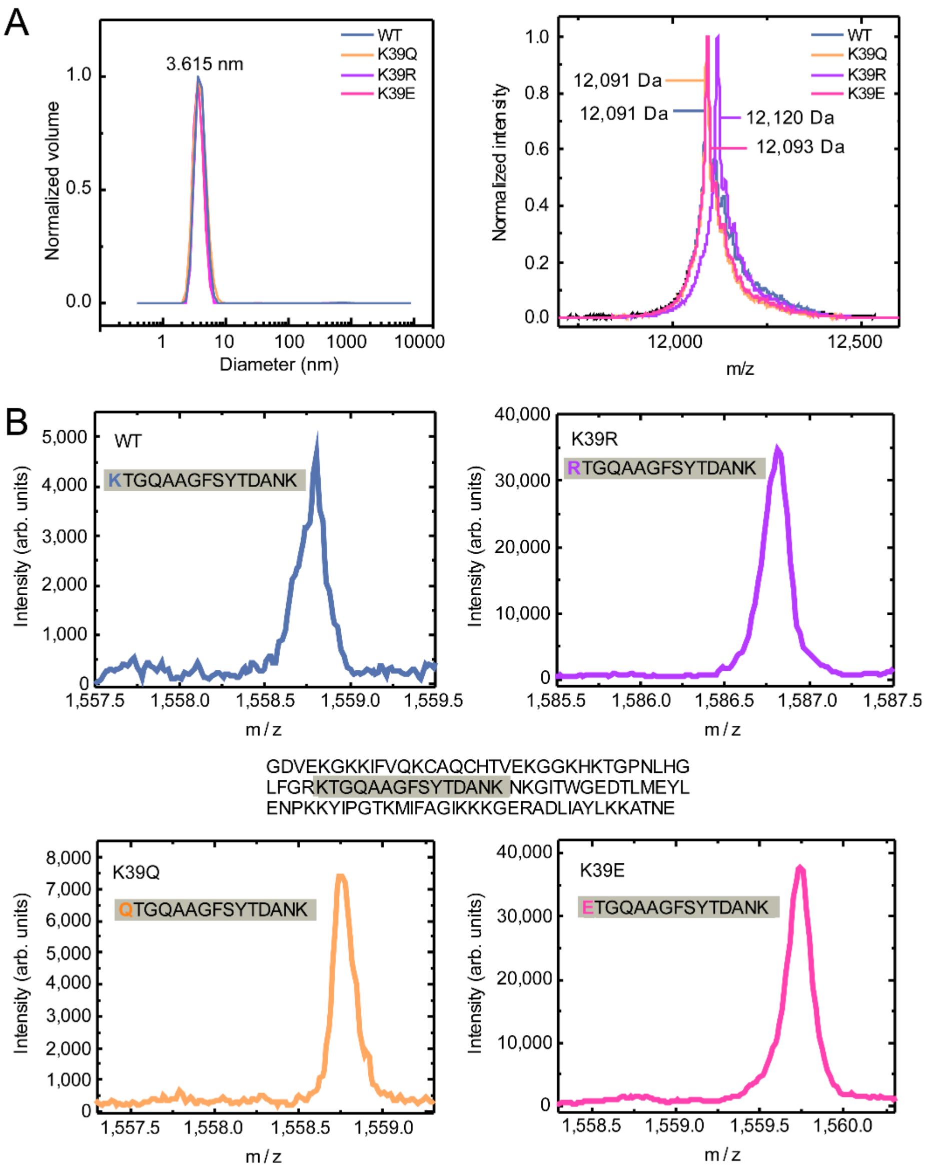

Supplementary Figure 4. Monomerization state and mass spectra of WT and mutant species of Cyt*c*. (A) Dynamic light scattering analysis (*left* panel) and MALDI-TOF spectra (*right* panel) of WT and Cyt*c* mutants. The molecular mass of WT and K39Q is 12,091 Da and of K39R and K39E Cyt*c* are 12,120 and 12,093 Da, respectively. **(B)** Tryptic digestion of proteins extracted from SDS-PAGE bands. The calculated masses of KTGQAAGFSYTDANK, QTGQAAGFSYTDANK, RTGQAAGFSYTDANK and ETGQAAGFSYTDANK fragments are 1,558.67, 1,558.62, 1,586.68 and 1,559.61 Da, respectively. Source data are provided as a Source Data file.

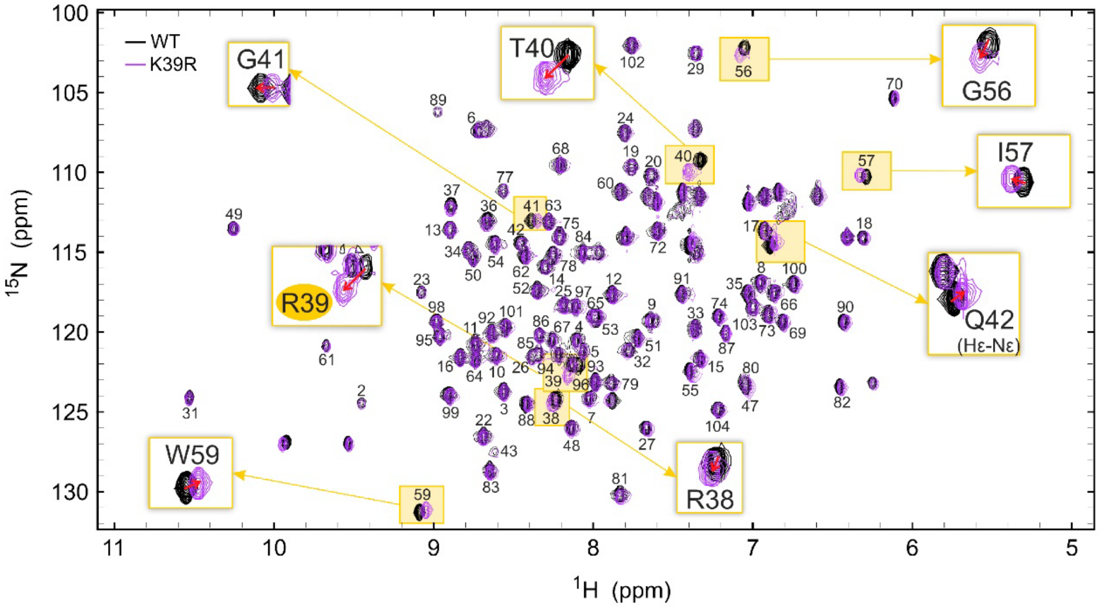

Supplementary Figure 5. Superimposition of 2D ^{1}H–^{15}N HSQC NMR spectra of WT (black) and K39R (purple) ^{15}N-labelled Cyt*c*. Insets: Zooms of the signals framed in yellow boxes. The red arrows stand for the chemical shift perturbations of the recombinant K39R protein as compared with the WT species. The yellow circle denotes the mutated residue.

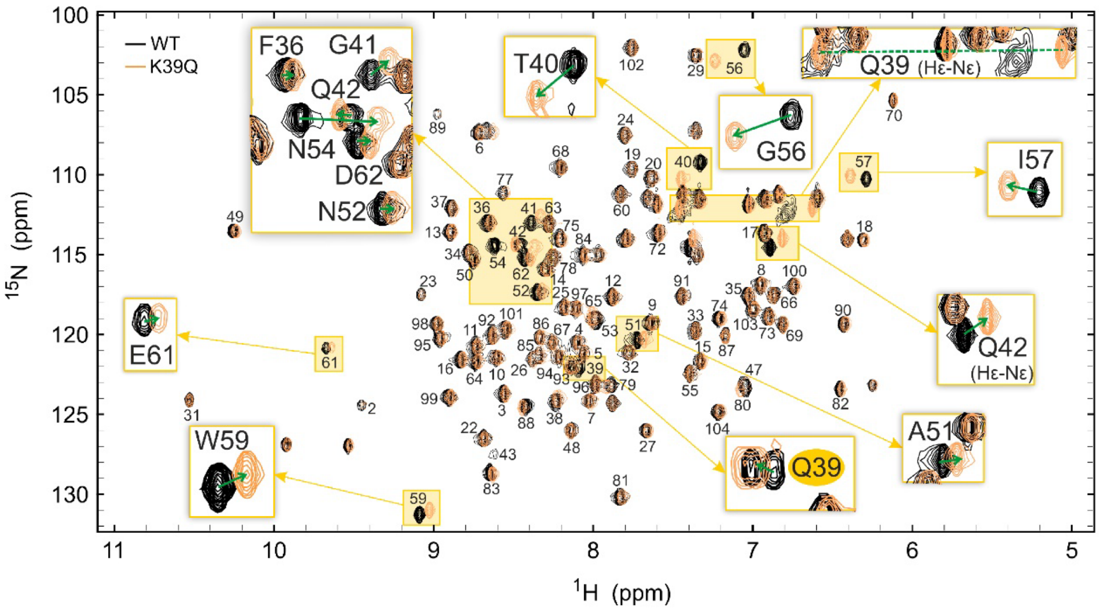

Supplementary Figure 6. Superimposition of 2D ^{1}H–^{15}N HSQC NMR spectra of WT (black) and K39Q (yellow) ^{15}N-labelled Cyt*c*. Insets: Zooms of the signals framed in yellow boxes. The green arrows stand for the chemical shift perturbations of the recombinant K39Q protein as compared with the WT species. The green dotted line shows the ^{15}N chemical shift at which the ^{1}H of the K39Q ε amine group appears. The yellow circle denotes the mutated residue.

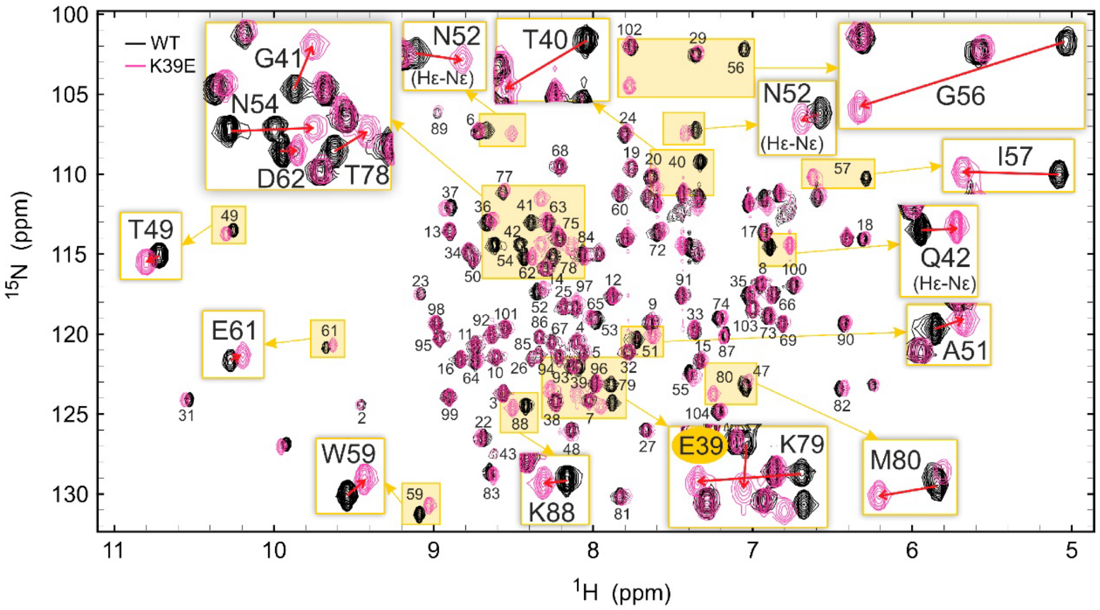

Supplementary Figure 7. Superimposition of 2D ^{1}H–^{15}N HSQC NMR spectra of WT (black) and K39E (pink) ^{15}N-labelled Cyt*c*. Insets: Zooms of the signals framed in yellow boxes. The red arrows stand for the chemical shift perturbations of the recombinant K39E protein as compared with the WT species. The yellow circle denotes the mutated residue.

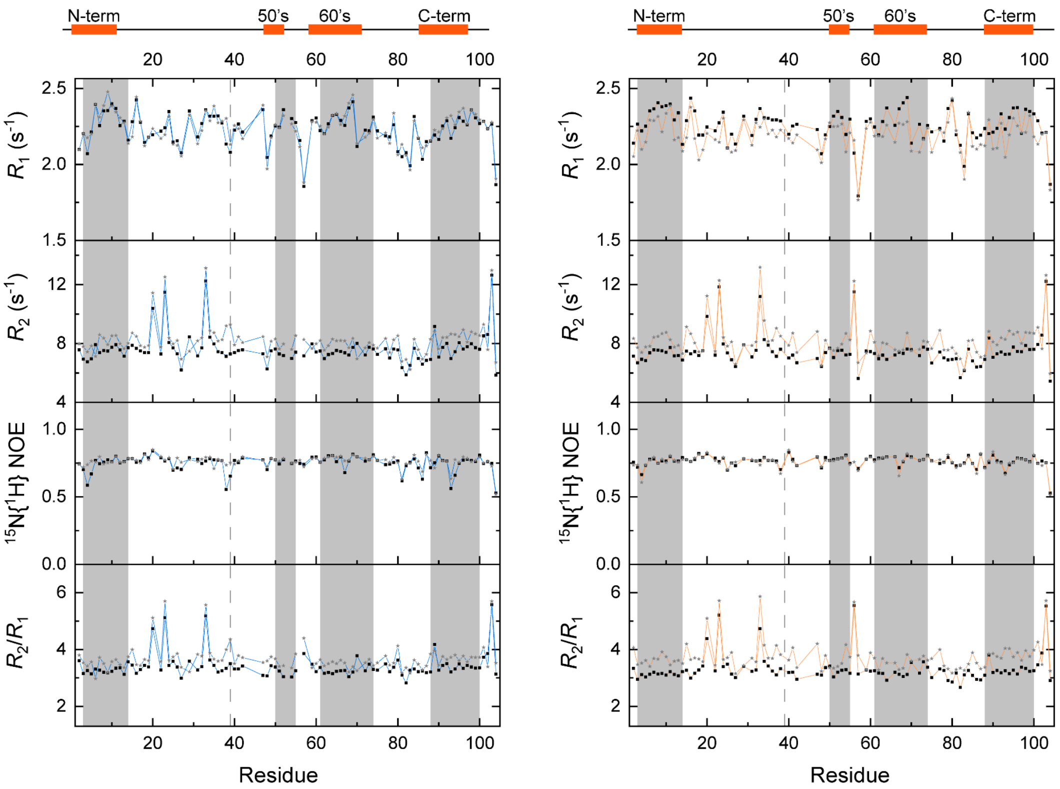

Supplementary Figure 8. Experimental data of the relaxation parameters of WT and K39Q Cyt*c*. Longitudinal relaxation rate R_1 and transversal relaxation rate R_2 (top), and heteronuclear NOE and R_2/R_1 (bottom) experimental values at 500 MHz for the reduced forms of WT (*left* panel, blue) and K39Q (*right* panel, yellow) Cyt*c*, plotted as a function of the residue number (n = 2). Data obtained from two relaxation measurements recorded on two biologically independent samples (WT or K39Q Cytc) are plotted with square and star dots. The errors reported are the standard errors derived from the fitting of the experimental data to an exponential decay. A scheme of the secondary structure elements is included at the top and the mutated position is indicated with dashed lines. Source data are provided as a Source Data file.

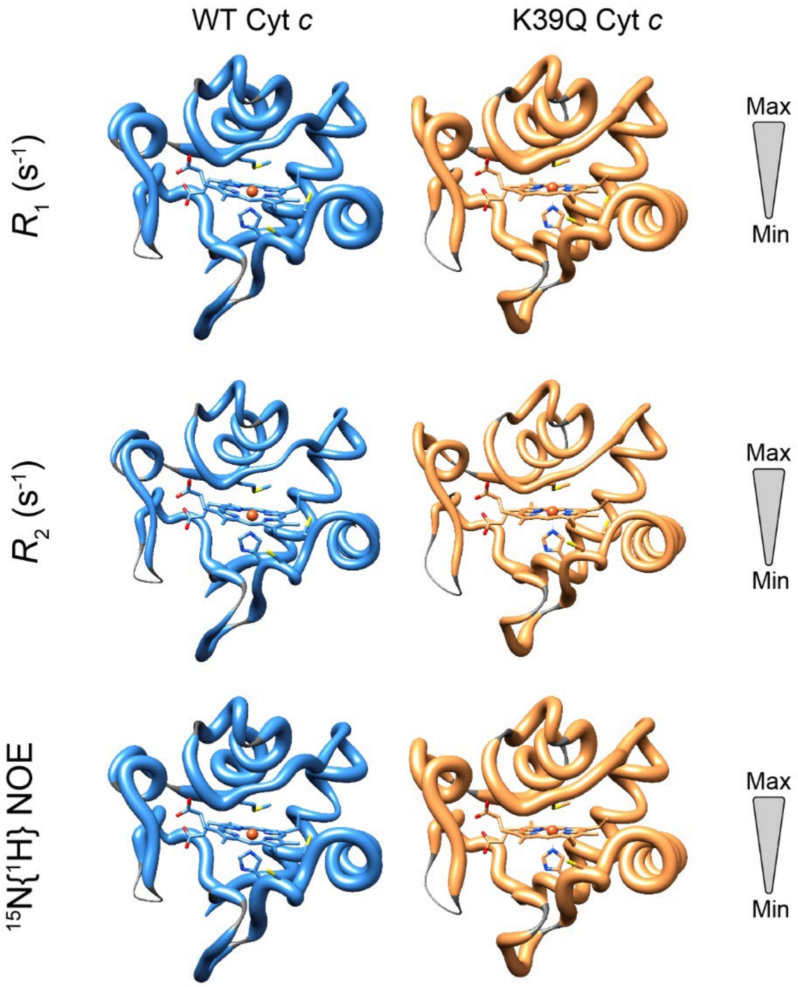

Supplementary Figure 9. NMR relaxation measurements and dynamic properties of WT and K39Q Cyt*c*. Sausage plot for WT (*left*) and K39Q (*right*) Cyt*c* rendered according to the relaxation properties (R_1, R_2 and heteronuclear NOE) of each residue. The thickness is directly related to the magnitude of the relaxation parameter. Undetectable or overlapping backbone resonances are in gray.

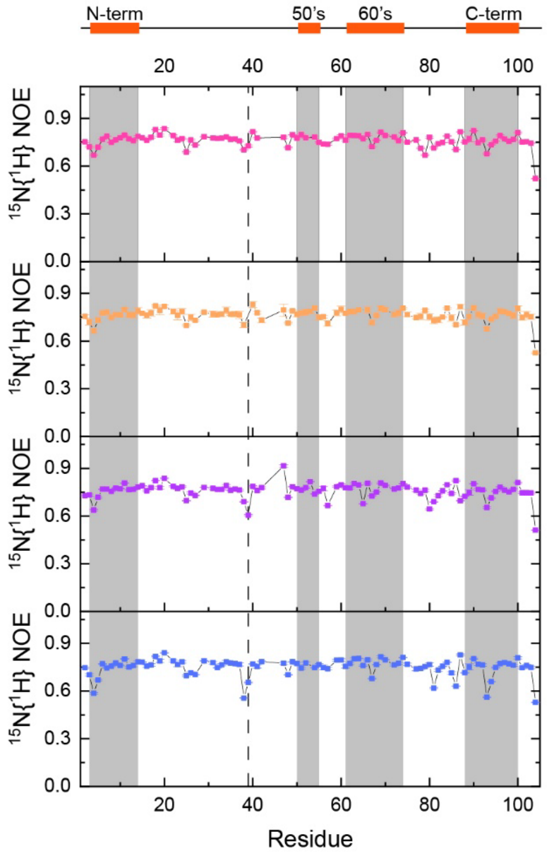

Supplementary Figure 10. Heteronuclear NOE NMR measurements of Cyt*c* mutants. Heteronuclear NOE ($^{15}N\{^1H\}$ NOE) experimental values at 500 MHz for the reduced forms of K39E (pink), K39Q (yellow), K39R (purple), and WT (blue) Cyt*c*, plotted as a function of the residue number. A scheme of the secondary structure elements is included at the top and the mutated position is indicated with dashed lines. Source data are provided as a Source Data file.

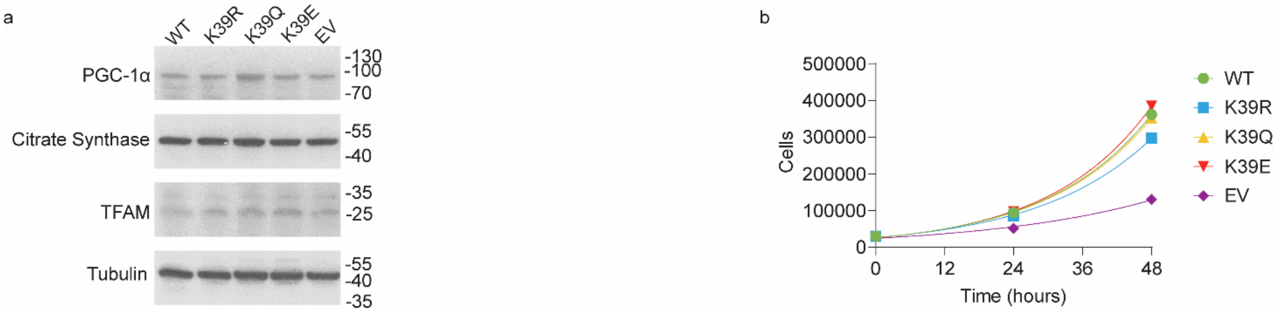

Supplementary Figure 11. Markers of mitochondrial biogenesis and growth curves for Cyt*c* variant expressing cell lines. (A) Representative western blot of Cyt*c* double knockout cells stably transfected with EV, WT, K39R, K39Q, and K39E constructs immunoprobed for PGC-1α, citrate synthase, TFAM, and loading control tubulin (n = 2). **(B)** Growth rate for the various cell lines (n = 6).

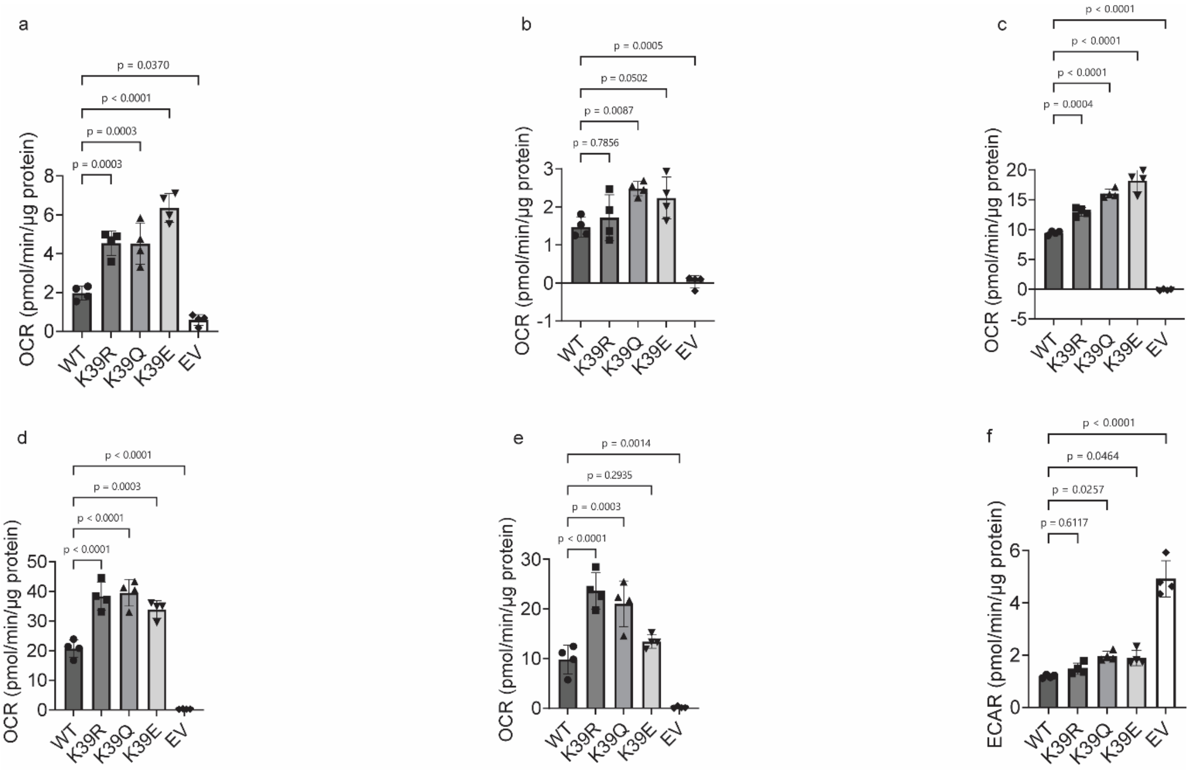

Supplementary Figure 12. Mitochondrial stress test parameters and extracellular acidification rate. Non-mitochondrial oxygen consumption **(A)**, proton leak **(B)**, ATP-coupled respiration **(C)**, maximal respiration **(D)**, spare respiratory capacity **(E)**, and baseline ECAR **(F)** in cells stably expressing WT, K39R, K39Q, K39E Cyt*c*, and EV (n = 4). Data are represented as means ± standard deviation.

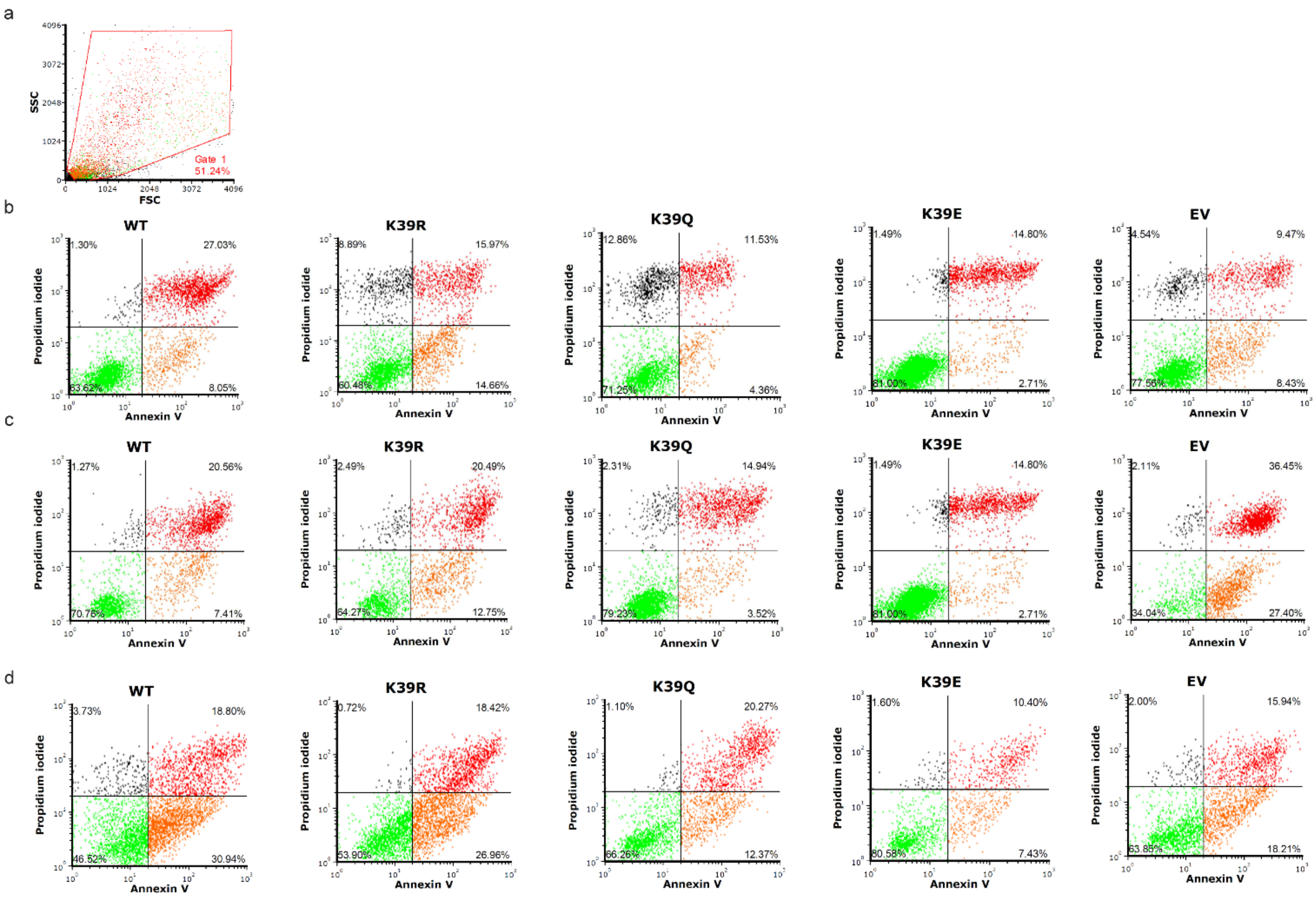

Supplementary Figure 13. Representative flow cytometry gating strategy. (A) Representative gating strategy for flow cytometry experiments. Representative flow cytometry scatter plots for live (green, unstained), early apoptotic (orange, positive only for annexin V), necrotic (black, positive only for propidium iodide), and late apoptotic cells (red, double-positive for both annexin V and propidium iodide) in cells stably expressing WT, K39R, K39Q, K39E Cyt*c*, and EV after exposure to 400 μM H_2O_2 for 16 h (n = 3) **(B)**, 16 h of oxygen-glucose deprivation followed by 1 h of reoxygenation (n = 3) **(C)**, or 1 mM thapsigargin for 24 h (n = 3) **(D)**.

Supplementary Table 1. Crystallographic data summarizing and comparing the structures of rodent somatic Cyt*c* (native), rodent somatic acetylmimetic Cyt*c* (K39Q), and porcine acetylated somatic Cyt*c*.

**STRUCTURE**	**WT Cyt*c* Mouse**	**Acetylmimetic Cyt*c* (K39Q) Mouse**	**Acetylated Cyt*c* Porcine**
**PDB CODE**	5C0Z	8DZL	8DVX
**DATA CODE**	141118x21a	220311x03c	201203x12a
**CRYSTALLIZATION**			
**Iron**	Oxidized	Oxidized	Oxidized
**Protein**	15 mg/mL Cyt*c* + 5 mM $K_3Fe(CN)_6$ in water	15 mg/mL Cyt*c* + 5 mM $K_3Fe(CN)_6$ in water	8 mg/mL Cyt*c* + 5 mM $K_3Fe(CN)_6$ in water
**Well**	25% PEG 4K, 8% isopropanol, 0.1 M Na Acetate, pH 6.5	Wizard Cryo 2#30: 40% PEG 600, 100 mM sodium citrate, pH 5.5	JBS3A2: 10% PEG 4K, 20% isopropanol, pH 5.0
**Drop**	1:1 Protein:Well	1:1 Protein:Well	1:1 Protein:Well
**Cryoprotectant**	30% PEG 4K, 8% isopropanol, 0.1 M sodium acetate, 20% ethylene glycol, 10 min soak with 5 mM $K_3Fe(CN)_6$, final pH 6.5	None – the well solution is also the cryoprotectant	15% PEG 4K, 20% isopropanol, 5 mM $K_3Fe(CN)_6$, 20% ethylene glycol, pH 5.0
**CRYSTAL DATA**			
**Space group:**	P1	P2(1)2(1)2(1)	P1
**Unit cell: a b c (Å)**<br>**α β γ (°)**	34.4 52.5 61.6<br>110.0 92.8 92.0	33.6 61.2 186.1<br>90.0 90.0 90.0	34.7 54.6 61.8<br>66.0 87.4 84.1
**Chains per A.U.**	4	4	4
**Matthews Coeff**	2.24	2.04	2.27
**Solvent %**	45.12	40.78	48.90
**X-RAY DATA**			
**Resolution (Å)**	49.228 - 1.124 (1.127 – 1.124)	58.10 - 1.36 (1.43 – 1.36)	49.61 - 1.50 (1.58 – 1.50)
**Beamline**	APS 21-ID-F	APS 21-ID-D	APS 21-ID-D
**Wavelength (Å)**	0.97872	1.12713	1.12713
**Reflections**	127840	83395	56854
**Completeness**	87.4 (44.7)	99.8 (99.9)	86.9 (80.6)
**Average I/sigma**	14.4 (2.0)	13.0 (2.4)	8.80 (2.0)
**Redundancy**	3.9 (3.7)	12.1 (12.5)	3.5 (3.1)
**Rmerge**	0.050 (0.548)	0.088 (0.797)	0.078 (0.532)
**REFINEMENT**			

**Rfactor**	0.132 (0.239)	0.141 (0.207)	0.171 (0.307)
**Rfree**	0.159 (0.240)	0.180 (0.253)	0.204 (0.360)
**Avg B-factor (Å2)**	15.97	23.60	23.74
**Protein atoms per A.U.**	3228	3228	3268
**Water molecules**	537	484	305
**Bond RMSD**	0.017	0.016	0.017
**Angle RMSD**	1.903	1.466	1.578
**$K_3Fe(CN)_6$ MOLECULES**	6	0	7
**HEME LINKS: Average (Å)**			
**C14 SG - HEM CAB**	1.90 (0.04)	1.76 (0.01)	1.77 (0.01)
**C17 SG - HEM CAC**	1.98 (0.04)	1.83 (0.01)	1.82 (0.00)
**H18 NE2 - FE2 (Å)**	2.02 (0.01)	1.97 (0.03)	2.02 (0.03)
**M80 SD - FE2 (Å)**	2.30 (0.01)	2.30 (0.03)	2.30 (0.04)

Supplementary Table 2. Mass spectrometry occupancy results reported in %.

	Control TA Samples						Ischemic TA Samples					
	1	2	3	4	5	6	1	2	3	4	5	6
K27	0.11		0.29					0.55		0.12	0.11	
K39							0.24	0.04		0.83		
K79			0.65							0.57		
K86	0.50		0.44							0.21	0.55	

Note: In the raw mass spectrometry results, lysines were numbered including the start methionine (mature Cyt*c* lacks the start methionine), therefore the numbering will be one higher than what is reported here in the final manuscript text.
