# Supplementary material for: Cytochrome c lysine acetylation regulates cellular respiration and cell death in ischemic skeletal muscle
Source: Nat Commun. 2023 Jul 13;14:4166. doi: 10.1038/s41467-023-39820-8 (PMC10345088; doi:10.1038/s41467-023-39820-8)
Supplement: Supplementary file 3 — Reporting summary [file 41467_2023_39820_MOESM3_ESM.pdf]

nature portfolio

Corresponding author(s): Maik Hüttemann, Irene Díaz-Moreno

Last updated by author(s): Jun 19, 2023

Reporting Summary

Nature Portfolio wishes to improve the reproducibility of the work that we publish. This form provides structure for consistency and transparency in reporting. For further information on Nature Portfolio policies, see our Editorial Policies and the Editorial Policy Checklist.

Statistics

For all statistical analyses, confirm that the following items are present in the figure legend, table legend, main text, or Methods section.

n/a	Confirmed	
☐	☒	The exact sample size (*n*) for each experimental group/condition, given as a discrete number and unit of measurement
☐	☒	A statement on whether measurements were taken from distinct samples or whether the same sample was measured repeatedly
☐	☒	The statistical test(s) used AND whether they are one- or two-sided<br>*Only common tests should be described solely by name; describe more complex techniques in the Methods section.*
☒	☐	A description of all covariates tested
☐	☒	A description of any assumptions or corrections, such as tests of normality and adjustment for multiple comparisons
☐	☒	A full description of the statistical parameters including central tendency (e.g. means) or other basic estimates (e.g. regression coefficient) AND variation (e.g. standard deviation) or associated estimates of uncertainty (e.g. confidence intervals)
☐	☒	For null hypothesis testing, the test statistic (e.g. *F*, *t*, *r*) with confidence intervals, effect sizes, degrees of freedom and *P* value noted<br>*Give P values as exact values whenever suitable.*
☒	☐	For Bayesian analysis, information on the choice of priors and Markov chain Monte Carlo settings
☒	☐	For hierarchical and complex designs, identification of the appropriate level for tests and full reporting of outcomes
☒	☐	Estimates of effect sizes (e.g. Cohen's *d*, Pearson's *r*), indicating how they were calculated

Our web collection on statistics for biologists contains articles on many of the points above.

Software and code

Policy information about availability of computer code

Data collection	COX activity data were recorded and analyzed using Oxytrace+ v1.0.48graph software (Hansatech). Crystal structures were solved using Phenix Program Suite, Phaser, AutoBuild, Phenix.Refine, PDB-REDO, YASARA version 20-07-04, and Molecule Operating Environment (MOE). Electrostatic surface potentials were calculated using UCSF Chimera 1.15 software (University of California; San Francisco, CA, USA) and the TLEAP module of AmberTools 2021 (University of California). Nuclear magnetic resonance analyses were processed using NMRPipe 10.9.
Data analysis	Mass spectrometry data were analyzed using MaxQuant software (v2.2.0.0) searched against the Sus domesticus proteome database UniProtKB (December 2016). Nuclear magnetic resonance data were analyzed with NMRDraw 10.9, TENSOR 2.0, and ROTDIF 1.1. Flow cytometry data were analyzed using FCS Express 7 software (De Novo Software; Glendale, CA, USA). Statistical analyses of the data were performed using Graphpad Prism v9.4.1 (Graphpad Software; San Diego, CA, USA). Microsoft Excel 2304 was used to generate Supplementary Figures 1D, 11B.

For manuscripts utilizing custom algorithms or software that are central to the research but not yet described in published literature, software must be made available to editors and reviewers. We strongly encourage code deposition in a community repository (e.g. GitHub). See the Nature Portfolio guidelines for submitting code & software for further information.

Data

Policy information about availability of data

All manuscripts must include a data availability statement. This statement should provide the following information, where applicable:

- Accession codes, unique identifiers, or web links for publicly available datasets
- A description of any restrictions on data availability
- For clinical datasets or third party data, please ensure that the statement adheres to our policy

The mass spectrometry data generated in this study have been deposited in the PRIDE repository under accession code PXD040915 [https://doi.org/10.6019/PXD040915]. The Sus domesticus proteome database UniProtKB with the ID UP000008227 [https://www.uniprot.org/uniprotkb/?query=proteome:UP000008227] and 26,104 entries (December 2016) was searched. The crystallography data generated in this study are available in the PDB repository with the identifiers 8DZL [https://www.rcsb.org/structure/8DZL] and 8DVX [https://www.rcsb.org/structure/8DVX]. The remaining data generated in this study are provided in the Supplementary Information/Source Data file.

Research involving human participants, their data, or biological material

Policy information about studies with human participants or human data. See also policy information about sex, gender (identity/presentation), and sexual orientation and race, ethnicity and racism.

Reporting on sex and gender	Human participants/data were not used.
Reporting on race, ethnicity, or other socially relevant groupings	Human participants/data were not used.
Population characteristics	Human participants/data were not used.
Recruitment	Human participants/data were not used.
Ethics oversight	Human participants/data were not used.

Note that full information on the approval of the study protocol must also be provided in the manuscript.

Field-specific reporting

Please select the one below that is the best fit for your research. If you are not sure, read the appropriate sections before making your selection.

☒ Life sciences ☐ Behavioural & social sciences ☐ Ecological, evolutionary & environmental sciences

For a reference copy of the document with all sections, see nature.com/documents/nr-reporting-summary-flat.pdf

Life sciences study design

All studies must disclose on these points even when the disclosure is negative.

Sample size	No sample-size calculation was performed. Generally, the minimum sample size used was 3 in order to perform statistical tests. The maximum sample size was 9 for mitoSOX and JC-10 experiments which were selected based on previous experience in our lab as the minimum sample size needed to achieve statistical significance (see PMID: 32781572). Individual data points are shown when possible with size constraints.
Data exclusions	No data were excluded from analyses.
Replication	Gels and blots were replicated (n = 3) as specified in the manuscript. NMR relaxation parameters were calculated based on two spectra, both of which are reported in the manuscript. Protein (COX, Caspase activity, rate of oxidation, superoxide assay, redox potential, heme degradation, and cardiolipin peroxidase assay) and cellular (ATP assay, Seahorse, JC-10, MitoSOX, OGD/R, and FACS) experiments were performed a second time under identical conditions to ensure the same general trend with the data. All attempts at replication were successful.
Randomization	Tibialis anterior muscle samples were selected to be control or ischemic. After harvesting the tissues, the tissues were cut and placed into air tight zip lock bags. A second researcher then randomly took the bags and either flash froze them (control TA) or exposed them to up to 1 hour of ischemic conditions in a water bath at 37°C (ischemic TA). Otherwise, randomization was not performed as experiments were quantified by the indicated software in an unbiased manner. There were no covariates that needed to be accounted for statistically. This is, in part, due to the fact that all protein samples and cells were treated identically for each experimental group.
Blinding	Initial mass spectrometry analyses were performed blinded to the experimental conditions (control or ischemic tibialis anterior muscle). Otherwise investigators were not blinded to experimental conditions as experiments were quantified by the indicated software in an unbiased manner.

Reporting for specific materials, systems and methods

We require information from authors about some types of materials, experimental systems and methods used in many studies. Here, indicate whether each material, system or method listed is relevant to your study. If you are not sure if a list item applies to your research, read the appropriate section before selecting a response.

Materials & experimental systems

n/a	Involved in the study
☐	☒ Antibodies
☐	☒ Eukaryotic cell lines
☒	☐ Palaeontology and archaeology
☐	☒ Animals and other organisms
☒	☐ Clinical data
☒	☐ Dual use research of concern
☒	☐ Plants

Methods

n/a	Involved in the study
☒	☐ ChIP-seq
☐	☒ Flow cytometry
☒	☐ MRI-based neuroimaging

Antibodies

Antibodies used

All antibodies used are listed in the methods section along with supplier name, catalog number, and dilution used:
rabbit anti-acetyl-lysine conjugated to horseradish peroxidase secondary antibody (#6952S, Lot #2, Cell Signaling Technology; Danvers, Massachusetts, USA);
mouse anti-Cytc antibody (#556433, Lot #8213785, Clone #7H8.2C12, BD Pharmingen; San Jose, CA, USA);
sheep anti-mouse IgG conjugated to horseradish peroxidase secondary antibody (#NA931V, Lot #15273046, GE Healthcare);
mouse anti-GAPDH antibody (#60004-1-Ig, Lot #10004129, Proteintech; Rosemont, IL, USA);
rabbit anti-PGC-1 antibody (#PA5-72948, Lot #XL3782595, Invitrogen; Carlsbad, CA, USA);
rabbit anti-citrate synthase antibody (#D7V8B, Lot #2, Cell Signaling Technology);
rabbit anti-tubulin antibody (#11224-1-AP, Lot #00016610, Proteintech);
mouse anti-TFAM antibody (#MA5-16148, Lot #XL3781443, Clone #18G102B2E11, Invitrogen);
donkey anti-rabbit IgG conjugated to horseradish peroxidase secondary antibody (#NA934V, Lot #14879061, GE Healthcare);
rabbit anti-Apaf-1 antibody (#8969, Lot #2, Cell Signaling Technology);
mouse anti-caspase-9 antibody (#9508S, Lot #7, Cell Signaling Technology);

Validation

All antibodies used are commercially available and have been validated by the respective manufacturer:

rabbit anti-acetyl-lysine conjugated to horseradish peroxidase secondary antibody (#6952S, Lot #2, Cell Signaling Technology; Danvers, Massachusetts, USA)
https://www.cellsignal.com/products/antibody-conjugates/acetylated-lysine-ac-k-100-multimab-rabbit-mab-mix-hrp-conjugate/6952

mouse anti-Cytc antibody (#556433, Lot #8213785, Clone #7H8.2C12, BD Pharmingen; San Jose, CA, USA)
https://www.bdbiosciences.com/en-us/products/reagents/western-blotting-and-molecular-reagents/western-blot-reagents/purified-mouse-anti-cytochrome-c.556433

sheep anti-mouse IgG conjugated to horseradish peroxidase secondary antibody (#NA931V, Lot #15273046, GE Healthcare)
https://www.cytivalifesciences.com/en/us/shop/protein-analysis/blotting-and-detection/blotting-standards-and-reagents/amersham-ecl-hrp-conjugated-antibodies-p-06260#tech-spec-table

mouse anti-GAPDH antibody (#60004-1-Ig, Lot #10004129, Proteintech; Rosemont, IL, USA)
https://www.ptglab.com/products/GAPDH-Antibody-60004-1-Ig.htm

rabbit anti-PGC-1 antibody (#PA5-72948, Lot #XL3782595, Invitrogen; Carlsbad, CA, USA)
https://www.thermofisher.com/antibody/product/PGC1-alpha-Antibody-Polyclonal/PA5-72948

rabbit anti-citrate synthase antibody (#D7V8B, Lot #2, Cell Signaling Technology)
https://www.cellsignal.com/products/primary-antibodies/citrate-synthase-d7v8b-rabbit-mab/14309

rabbit anti-tubulin antibody (#11224-1-AP, Lot #00016610, Proteintech)
https://www.ptglab.com/products/TUBA1B-Antibody-11224-1-AP.htm

mouse anti-TFAM antibody (#MA5-16148, Lot #XL3781443, Clone #18G102B2E11, Invitrogen)
https://www.thermofisher.com/antibody/product/TFAM-Antibody-clone-18G102B2E11-Monoclonal/MA5-16148

donkey anti-rabbit IgG conjugated to horseradish peroxidase secondary antibody (#NA934V, Lot #14879061, GE Healthcare)
https://www.cytivalifesciences.com/en/us/shop/protein-analysis/blotting-and-detection/blotting-standards-and-reagents/amersham-ecl-hrp-conjugated-antibodies-p-06260#tech-spec-table

rabbit anti-Apaf-1 antibody (#8969, Lot #2, Cell Signaling Technology)
https://www.cellsignal.com/products/primary-antibodies/apaf-1-d5c3-rabbit-mab/8969

mouse anti-caspase-9 antibody (#9508S, Lot #7, Cell Signaling Technology)
https://www.cellsignal.com/products/primary-antibodies/caspase-9-c9-mouse-mab/9508

rabbit anti-caspase-3 antibody (#14220S, Lot #4, Cell Signaling Technology)
https://www.cellsignal.com/products/primary-antibodies/caspase-3-d3r6y-rabbit-mab/14220

Eukaryotic cell lines

Policy information about cell lines and Sex and Gender in Research

Cell line source(s)	Cytochrome c double knockout mouse lung fibroblast cell line was sourced from the lab of Dr. Carlos Moraes at the University of Miami as described in PMID: 17210651.
Authentication	The original Cytochrome c double knockout mouse lung fibroblast cell line was sourced directly from Dr. Carlos Moraes at the University of Miami. Validation was performed in PMID: 17210651 as well as by us to confirm the absence of Cytochrome c. Successful transfection with recombinant plasmids were confirmed via sequencing.
Mycoplasma contamination	Cells were routinely tested for mycoplasma contamination every 6 months. All cells used for experiments in this study tested negative for mycoplasma before and after experiments were performed.
Commonly misidentified lines (See ICLAC register)	No commonly misidentified cell lines were used in this study.

Animals and other research organisms

Policy information about studies involving animals; ARRIVE guidelines recommended for reporting animal research, and Sex and Gender in Research

Laboratory animals	Domestic farm swine were used in this study.
Wild animals	No wild animals were used in this study.
Reporting on sex	Both male and female pigs were used in this study, with the results being reported in the results section separated by gender.
Field-collected samples	No field-collected samples were used in this study.
Ethics oversight	All animal tissues were sourced from Wayne State University School of Medicine (Detroit, MI, USA) as discarded tissues with institutional animal care and use committee approval under protocol IACUC-18-11-0859.

Note that full information on the approval of the study protocol must also be provided in the manuscript.

Flow Cytometry

Plots

Confirm that:

☒ The axis labels state the marker and fluorochrome used (e.g. CD4-FITC).

☒ The axis scales are clearly visible. Include numbers along axes only for bottom left plot of group (a 'group' is an analysis of identical markers).

☒ All plots are contour plots with outliers or pseudocolor plots.

☒ A numerical value for number of cells or percentage (with statistics) is provided.

Methodology

Sample preparation	Cells (1 x 10^6) were seeded on 10 cm cell culture dishes and cultured overnight in growth media. The next day, cells were exposed to either H2O2 (400 µM for 16 h), or OGD/R (16 h oxygen-glucose deprivation/1 h reoxygenation) as described above, or thapsigargin (#328570050, Thermo Fisher Scientific) (1 mM for 24 h). After 48 h from the initial seeding, cells were trypsinized, washed twice with 1x PBS, counted, and a total of 1 x 10^6 cells were resuspended in 1 mL 1x annexin V binding buffer from the FITC annexin V apoptosis detection kit I (#556547; RRID: AB_2869082; BD Pharmingen). A total of 450 µL cell suspension was incubated for 15 min with 6 µL annexin V and 6 µL PI.
Instrument	Data were collected using a CyFlow Space flow cytometer (Sysmex America, Inc.; Lincolnshire, IL, USA).
Software	The results were analyzed using FCS Express 7 software (De Novo Software; Glendale, CA, USA).
Cell population abundance	Gating strategy is shown in the supplemental figures. Cells were not used for experiments following flow cytometry.

Gating strategy

FSC/SSC gates were used only to gate against clumped cells and to enrich for live cells. Staining gating was based on unstained controls or single stained positive controls. Examples of gating are included in the supplemental figures.

☒ Tick this box to confirm that a figure exemplifying the gating strategy is provided in the Supplementary Information.
